# Supplementary material for: The effects of genital myiasis on the diversity of the vaginal microbiota in female Bactrian camels
Source: BMC Vet Res. 2022 Mar 5;18:87. doi: 10.1186/s12917-022-03189-5 (PMC8897907; doi:10.1186/s12917-022-03189-5)
Supplement: Supplementary file 5 — Additional file 5. [file 12917_2022_3189_MOESM5_ESM.zip › MPL201709200_16s_yy/Treat1/C07_picrust/heatmap.pdf]

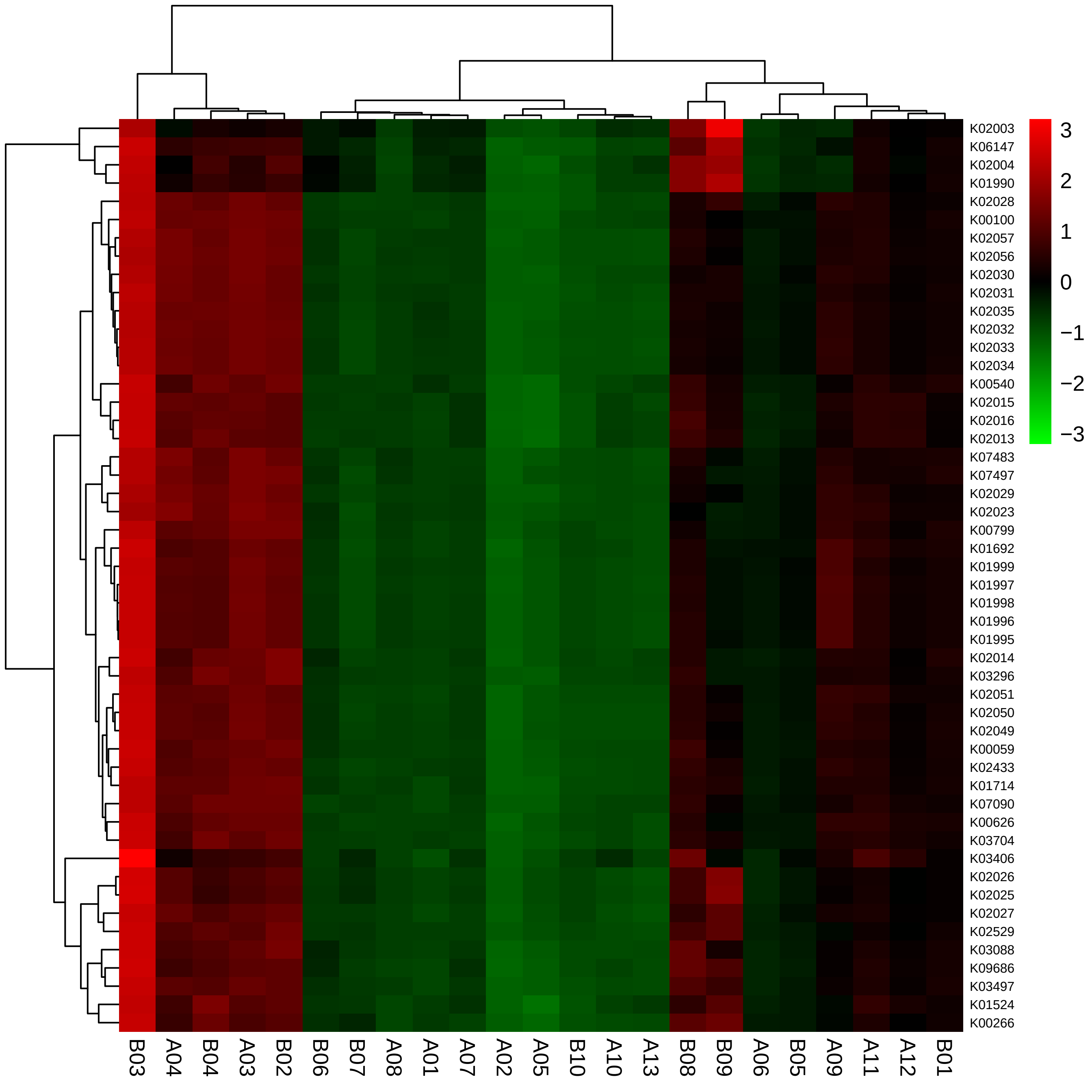

K02003
K06147
K02004
K01990
K02028
K00100
K02057
K02056
K02030
K02031
K02035
K02032
K02033
K02034
K00540
K02015
K02016
K02013
K07483
K07497
K02029
K02023
K00799
K01692
K01999
K01997
K01998
K01996
K01995
K02014
K03296
K02051
K02050
K02049
K00059
K02433
K01714
K07090
K00626
K03704
K03406
K02026
K02025
K02027
K02529
K03088
K09686
K03497
K01524
K00266
B03
A04
B04
A03
B02
B06
B07
A08
A01
A07
A02
A05
B10
A10
A13
B08
B09
A06
B05
A09
A11
A12
B01
3
2
1
0
−1
−2
−3
